# Netmums: a phase II randomized controlled trial of a guided Internet behavioural activation treatment for postpartum depression

**DOI:** 10.1017/S0033291713002092

**Published:** 2013-10-09

**Authors:** H. A. O'Mahen, D. A. Richards, J. Woodford, E. Wilkinson, J. McGinley, R. S. Taylor, F. C. Warren

**Affiliations:** 1 Mood Disorders Centre, University of Exeter, Washington Singer Building, Exeter, UK; 2 University of Exeter Medical School, Washington Singer Building, Exeter, UK; 3 Academic Unit of Child and Adolescent Psychiatry, Imperial College, St Mary's Campus, Norfolk Place, LondonUK; 4 Netmums.com, Marylebone Business Centre, London, UK; 5 University of Exeter Medical School, Veysey Building, Exeter, UK

**Keywords:** Anxiety, behavioural activation, bonding, cognitive behavioural therapy, depression, Internet interventions, postnatal depression

## Abstract

**Background:**

Despite the high prevalence of postnatal depression (PND), few women seek help. Internet interventions may overcome many of the barriers to PND treatment use. We report a phase II evaluation of a 12-session, modular, guided Internet behavioural activation (BA) treatment modified to address postnatal-specific concerns [Netmums Helping With Depression (NetmumsHWD)].

**Method:**

To assess feasibility, we measured recruitment and attrition to the trial and examined telephone session support and treatment adherence. We investigated sociodemographic and psychological predictors of treatment adherence. Effectiveness outcomes were estimated with the Edinburgh Postnatal Depression Scale (EPDS), Generalized Anxiety Disorder-7, Work and Social Adjustment Scale, Postnatal Bonding Questionnaire, and Social Provisions Scale.

**Results:**

A total of 249 women were recruited via a UK parenting site, Netmums.com. A total of 83 women meeting DSM-IV criteria for major depressive disorder were randomized to NetmumsHWD (*n* = 41) or treatment-as-usual (TAU; *n* = 42). Of the 83 women, 71 (86%) completed the EPDS at post-treatment, and 71% (59/83) at the 6-month follow-up. Women completed an average of eight out of 12 telephone support sessions and five out of 12 modules. Working women and those with less support completed fewer modules. There was a large effect size favouring women who received NetmumsHWD on depression, work and social impairment, and anxiety scores at post-treatment compared with women in the TAU group, and a large effect size on depression at 6 months post-treatment. There were small effect sizes for postnatal bonding and perceived social support.

**Conclusions:**

A supported, modular, Internet BA programme can be feasibly delivered to postpartum women, offering promise to improve depression, anxiety and functioning.

## Introduction

The postpartum period is a critical developmental period for mothers and infants, during which an estimated 13% of women suffer from postpartum depression (Gavin *et al.* 2005). The impact of postpartum depression, which affects both the mother and infant, is significant. The effects of these early problems are profound, with longitudinal studies demonstrating that postpartum depression has long-term negative cognitive, social and emotional implications for the child (Pawlby *et al.* 2008; Murray *et al.* 2010). Postpartum depression recognition amongst health professionals is poor (Murray *et al.* 2004) and help-seeking amongst postpartum women remains low (17–25%; Buist *et al.* 2005). In recognition of this, both USA- and UK-based guidelines recommend prioritizing prompt intervention for postpartum depression (National Institute for Health and Clinical Excellence, 2007; National Research Council and Institute of Medicine, 2009), however, women face a number of factors that impede their ability to receive appropriate treatment.

The postpartum period presents specific barriers to accessing appropriate depression treatment. Women report a preference for psychotherapy over antidepressants, particularly when they are breastfeeding (Dennis & Chung-Lee, 2006). The postpartum period presents unique barriers to office-based treatment, and includes specific treatment-related needs (Dennis & Chung-Lee, 2006; O‘Mahen & Flynn, 2008; Goodman, 2009). Mothers of infants and young children report struggles with transportation and childcare (Goodman, 2009), while variable infant feeding and napping schedules can interfere with the ability of women to attend regularly scheduled appointments. Despite stigma-reduction campaigns aimed at improving awareness of postpartum depression (e.g. Crisp *et al.* 2005), women continue to report perceived stigma about postpartum depression (O'Mahen & Flynn 2008; Goodman, 2009). A significant minority of women also report fears that their children will be ‘taken away’ if health providers discover they suffer from depression (Dennis & Chung-Lee, 2006). In recent phase I (Craig *et al.* 2008) development work we have conducted, mothers experiencing postnatal depression (PND) also expressed concerns about treatments lacking content specific to the postpartum period (O'Mahen *et al.* 2012, 2013*a*). These included: difficulties in finding normalizing information about mothering; problems in accessing and utilizing support from others; maternal sleep difficulties associated with infant sleep schedules and problems adjusting to and managing the busy schedule of an infant balanced against other valued tasks. Finally, empirically supported psychotherapies are not equivalently distributed geographically, resulting in inequities in access to appropriate treatment (Payne & Myhr, 2010).

Internet delivery systems offer an alternative, promising approach that may circumvent many of the difficulties of face-to-face delivery techniques (Kohn *et al.* 2004). Offering therapy online could provide women with the flexibility of receiving treatment at a time and place convenient for them, avoid the time and economic costs associated with providing home-based therapy, and reduce concerns about confidentiality and delivery in home and public spaces. Internet-based interventions also provide a level of anonymity not possible with face-to-face therapy (e.g. distance-based, supporter does not see client's face) (Beattie *et al.* 2009), potentially overcoming women's concerns about stigma (Dennis & Chung-Lee, 2006). In support of this, a recent investigation of online screening for PND demonstrated that mothers were more likely to report sensitive information regarding their mood over the Internet (Le *et al.* 2009). Lastly, computerized interventions require less overall therapist contact time, thereby reducing costs and improving treatment accessibility (Andrews *et al.* 2010).

### Why create a new online treatment for postpartum depression?

Online cognitive behavioural therapy (CBT) for depression has been demonstrated in a number of randomized controlled trials (e.g. Christensen *et al.* 2004; Warmerdam *et al.* 2008; Meyer *et al.* 2009; Perini *et al.* 2009) and systematic reviews (e.g. Spek *et al.* 2007; Kaltenthaler *et al.* 2008). A meta-analysis of both supported and unsupported online interventions for major depression (Andrews *et al.* 2010) found moderate to large levels of effectiveness [effect size^1^† 0.78, 95% confidence interval (CI) 0.59–0.96].

However, recent literature suggests that online CBT programmes cannot always be seamlessly individualized for specific treatment conditions, thereby reducing the acceptability of programmes to individuals with particular conditions (Hind *et al.* 2009). Because our phase I work (O'Mahen *et al.* 2012) suggested that women suffering from postpartum depression required specific treatment adaptations, we developed an online behavioural activation (BA) treatment that was specifically adapted to the concerns of postnatal women. BA is a straightforward and parsimonious approach that contains a functional analytical approach to the behavioural components of CBT, without using the cognitive components, and has been shown to be as effective as traditional CBT (Jacobson *et al.* 2001; Dimidjian *et al.* 2006).

We previously tested the feasibility and initial efficacy of an 11-session minimal support version of online BA modified for PND (O'Mahen *et al.* 2013*b*). Although there were small to moderate treatment effect sizes for women who were trial adherent, there were high rates of attrition (63%). Women in the trial reported difficulties keeping up with the materials and struggling to individualize the materials. We modified the treatment materials to take a modular approach, and added a guided support component in order to improve treatment understanding and adherence rates (Cuijpers *et al.* 2010).

In accord with the UK Medical Research Council framework for complex interventions, we report the results of the subsequent phase II (Craig *et al.* 2008) randomized controlled trial of the guided BA-based modular computerized treatment programme that we developed for postpartum depression (Netmums Helping With Depression; NetmumsHWD), compared with treatment-as-usual (TAU). The aims of the study were: (1) to establish recruitment and trial adherence rates; (2) to determine treatment adherence and predictors of modules and telephone sessions; (3) to assess the preliminary effectiveness of NetmumsHWD on depressive and anxious symptoms, work and social impairment, perceived support, and maternal self-reported bonding with her infant in order to help inform future sample size calculations; and (4) to gather data on health care utilization at baseline and at post-treatment in preparation for a health economic assessment.

## Method

### Recruitment

We recruited participants between October 2011 and February 2012 by advertisements on website banners, ‘new “Helping with Depression” treatment study’, on the UK Netmums parenting website front page and on their PND chat room page (www.netmums.co.uk), through the Netmums' newsletter, via emails to women who had posted on the PND chat room in the previous 12 months, and via the Netmums Twitter and Facebook sites. Interested participants provided electronic informed consent, and then accessed information on the Netmums website describing the treatment, eligibility and randomization procedures. Consenting women filled in screening questionnaires online, including the Edinburgh Postnatal Depression Scale (EPDS), to assess their potential eligibility for the trial.

### Participants

Women completing the screening battery who were aged over 18 years, had given birth to a live baby in the last year, scored greater than 12 on the EPDS, did not experience substance abuse, psychosis, and spoke English were potentially eligible for the study. We contacted potentially eligible women via telephone to complete a diagnostic clinical assessment. Women who met International Classification of Diseases, 10th Revision (ICD-10) criteria for major depressive disorder (MDD) were eligible for the study.

### Randomization and allocation

Eligible and consenting women were randomized to receive either NetmumsHWD or TAU, minimized on depression severity (EPDS ⩾13) and whether or not they were currently receiving pharmacological treatment. The minimization algorithm included a stochastic element to inform the allocation process and was administered remotely using a computer-generated code to ensure concealment. Randomization occurred online; eligible women were sent an electronic link to a webpage where they could learn their randomization assignment.

### Interventions

#### Treatment (NetmumsHWD)

Following a previous trial (O'Mahen *et al.* 2013*b*) which developed the BA treatment from qualitative work with depressed postnatal women (O'Mahen *et al.* 2012), a core group of stakeholders (specialist health visitor, Netmums peer supporter, and two service users) worked with the researchers to modify the treatment content and presentation to fit with previous trial participants' feedback. The modified 12-session treatment course consisted of a core BA module (five sessions) and a relapse prevention session. Women also chose two optional modules from a list of a possible six (see Table 1 for a description of the treatment). All modules followed the BA functional analytical framework (Addis & Martell, 2004). The content included interactive exercises paired with extensive worked examples. Sessions could be supplemented by a cache of resources on the Netmums site, including their ‘meet a mum’ feature, which allowed women to connect with other women in their local area. Women also had access to a chat room that was moderated by peer supporters and was open only to women in the treatment. Presentation of the website was simplified to ensure that a wide range of users with varying technical skill could use the website without training.

**Table 1.** Description of session content

Module title	Session title	Session content
Core module	Behavioural activation	
Session 1	Understanding depression and the mood–activity link	Monitoring mood and activity
Session 2	How did I get here?	Introduction to avoidance and functional analysis (TRAP)
Session 3	‘Help! I want to get better!’ Moving out of depression	Introduction to planning alternative meaningful behaviour (TRAC)
Session 4	When the going gets tough. What to do	Contingency planning. Breaking down tasks and problem-solving
Session 5	Getting the balance right. Being a mother and a person	Achieving TRACs in creative and realistic ways. Prioritizing
Optional modules		
Optional module 1	Being a good enough mother	
Session 1	Introduction	Introduction to topic. Mother could choose one of three parenting topics to focus on within this module
Session 1, topics 1–3	Topic 1: Attachment and bonding with my baby. Understanding	Functional analysis of:
	TRAPs	Topic 1: play with baby
	Topic 2: Colicky, fussy baby	Topic 2: colicky, fussing situations with baby, including mother's reactions and coping
	Topic 3: Feeding difficulties	Topic 3: feeding difficulties
Session 2, topics 1–3	Getting back on TRAC	Exploring alternative specific behaviours in each topic specific area. (e.g. play behaviours, maternal self-soothing responses to colic). Incorporate specific Netmums tools where appropriate (chat rooms to brainstorm ideas, ideas for managing colic)
Session 3, topics 1–3	Building on successes and overcoming obstacles	Building depth in new TRACs, problem-solving when barriers encountered
Optional module 2	Support and communication module	
Session 1	Don't go it alone. Getting the support you need	Identifying specific difficult conversational patterns. Functional analysis of conversation. Picking specific conversations to address. Identifying alternative options (TRAC) with negative conversations
Session 2	Moving on to more difficult conversations	Building greater depth with more conversational TRACs
Session 3	Building a lifeline with other mothers	Improving communication and relationships with other mothers
Session 4	Continuing to build a lifeline with other mothers	Identifying conversation TRAPs from previous week. Breaking down problem conversations. Instituting new TRACs. Developing new TRAC conversation goals
Optional module 3	Changing roles and relationships (transitions)	
Session 1	Introduction	Introduce topic of transition and way it makes an impact on goals and mood. Identify at least one topic of focus
Session 1, topics 1–4	Topic 1: Work	Identify reinforcers in important goals pre-baby.
	Topic 2: Friendship and social independence	Identify current missing reinforcers (e.g. enjoyed a sense of accomplishment and recognition at work, missing this now). Identify opportunities to implement reinforcers in context of current life within TRAC model
	Topic 3: Relationship with partner	
	Topic 4: Always struggled! Was never a ‘good’ housewife and now I'm not a good mother either	
Session 2, topics 1–4	Overcoming barriers, Building on TRACs	Problem-solving barriers to TRAC, building on new TRACs in same domain when successful
Session 3, topics 1–4	Managing conflict between long-term goals. Linking short-term goals with restructured long-term goals	Identifying goal conflict (e.g. work and family). Brainstorming individual specific solutions. Linking short-term goals to long-term goal solutions
Optional module 4	Sleep	
Session 1	Understanding your sleeping patterns and setting goals	Functional analysis (TRAP) of maternal sleep patterns in context of infant sleep. Infant sleep difficulties referred to Netmums infant sleep clinic
Session 2	Developing healthy sleep behaviour	Sleep hygiene in the postpartum period + alternative behaviours to problematic sleep behaviours identified in functional analysis
Session 3	What to do when the going gets tough	Problem-solving difficulties with instituting healthy sleep behaviours
Optional module 5	‘Sticky thoughts’, rumination	
Session 1	Understanding ‘sticky thoughts’ and how you became stuck	Identify individual reasons for rumination, functional analysis of rumination. Identifying triggers for rumination
Session 2	Getting unstuck from ‘sticky thoughts’	Changing triggers, identifying modes of thinking ‘abstract’ to ‘concrete’
Session 3	What to do when the going gets tough	Problem-solving barriers to TRACs
Optional module 6		
Session 1	Understanding postnatal anxiety and learning to gently face your fears	Psychoeducation about psychophysiology of anxiety and reinforcing pattern of avoidance. Functional analysis of specific anxiety situations. Creating an anxiety hierarchy. Picking an exposure exercise for the week
Session 2	Facing your fears: building on success and dealing with obstacles and barriers	Problem-solving specific barriers to exposure
Session 3	Continuing to face your fears	Working up the hierarchy

TRAP, Trigger, Response, Avoidance Pattern; TRAC, Trigger, Response, Alternative Coping.

Women received weekly phone call support from mental health workers with undergraduate degrees and 1 year of further clinical qualification in psychological therapies under the UK Improving Access to Psychological Therapies (IAPT) training scheme. Supporters, who had previously been trained in ‘low-intensity’ (monitoring and scheduling) BA, received 5 days of training in the ‘high-intensity’ (functional analysis-based) perinatal-specific BA approach used in the treatment (Dimidjian *et al.* 2006; O'Mahen *et al.* 2013*b*). Training involved a mix of didactics and roleplay around conducting functional analysis in perinatal-specific domains with the chief investigator (H.O.), a clinical psychologist with specialty expertise in BA and perinatal depression, and an IAPT trainer (J.W.). Supporters provided telephone sessions of 20–30 min that focused on answering questions about treatment material and working through barriers to treatment implementation. Supporters had computer access to women's completed exercises. They followed sessional support guides that outlined key topic areas to cover for each session (e.g. functional analysis of infant–mother play, breaking down goals, brainstorming solutions). Sessions were audio-recorded and 20% of the sessions were monitored weekly for adherence to the sessional guides. Adherence was monitored and addressed in weekly 1-hour supervision with H.O., the chief investigator. Women who missed sessions were actively followed up by supporters using telephone calls, emails and text messages to reschedule appointments.

#### Control (TAU)

The TAU condition was allowed to vary according to usual practice. Women in both groups had access to Netmums' general depression chat room throughout the course of the study. This chat room is moderated by health visitors and parent supporters who provide email/chat room posting support and advice for depression.

### Measurement

#### Recruitment and attrition

We collected data on the numbers of potential and actual participants presenting at each stage of the recruitment and follow-up process to quantify the flow of participants through the trial and assess the feasibility, and recruitment and retention yield of our procedures. Data were collected online and via telephone.

#### Treatment adherence and predictors of adherence

We measured the numbers of modules viewed by women, modules completed, and the numbers of telephone support sessions received by participants. We collected demographic data at baseline on income level, work and relationship status, education qualifications, and number of children to assess the moderating effects of these variables.

#### Depressive symptoms

We assessed participants' mood symptoms at baseline, 17 weeks post-treatment, and 6 months post-treatment with the EPDS (Cox *et al.* 1987), a widely used, reliable and valid self-report 10-item scale measuring symptoms of PND (range 0–30).

#### Anxiety

We used the Generalized Anxiety Disorder Scale (GAD-7), a seven-item measure, to assess levels of general anxiety (Spitzer *et al.* 2006). The GAD-7 has been demonstrated to have good validity and reliability (Löwe *et al.* 2008) and accurately predicts the presence of anxiety disorders (Swinson, 2006).

#### Work and social impairment

We measured functional impairment with the Work and Social Adjustment Scale (WASAS; Mundt *et al.* 2002), a five-item measure that has demonstrated very good to excellent reliability and validity (Mundt *et al.* 2002). The WASAS has been used with women suffering from postpartum depression (Reay *et al.* 2006).

#### Social support

We assessed perceived availability of support with the 24-item Social Provision Scale (SPS; Cutrona & Russell, 1987). The SPS has good reliability and validity and has been used widely in studies of social support. Higher scores represent greater perceived support.

#### Postnatal bonding

We used the 25-item Postnatal Bonding Questionnaire (PBQ; Brockington *et al.* 2001) to assess parental perceptions of the relationship with the infant. The PBQ has adequate internal reliability and validity and is sensitive to changes in treatment. Higher scores represent greater reported difficulties in bonding.

#### Health service utilization

We used the Adult Service Use Schedule (AD-SUS), an instrument designed on the basis of previous studies of adult mental health populations (Byford *et al.* 2003). The AD-SUS asks recipients for the number and length of contacts with various services and professionals relevant to the disease of interest over the previous 3 months. We further adapted the AD-SUS to include services utilized by mothers of young infants.

### Sample size

Our primary intent was to estimate recruitment, retention and adherence levels for the intervention, and to estimate standard deviations for the primary outcome, the EPDS, to inform a sample size calculation for a future fully powered trial. We aimed to randomize approximately 80 participants in total. With an estimated 20% attrition rate, a sample size of 80 allowed us to estimate the attrition rate with a 95% CI width of approximately 20 percentile points. Further, with an estimated 20% attrition rate, a sample size of 64 is sufficient for estimation of retention and adherence levels and standard deviations of outcome variables (Julious, 2005).

### Statistical analysis

We analysed recruitment and retention data using counts of participants at each stage in recruitment and retention, expressed as a percentage of the total number of participants initially indicating interest and in relation to the preceding step in recruitment. We report means and standard deviations for treatment adherence. We used correlation, *t* tests, and Goodman and Kruskal lambda to analyse predictors of treatment adherence.

We analysed all clinical outcome data on an intention-to-treat basis. We examined demographic variables to assess pre-treatment balance across the groups and undertook descriptive analysis of baseline and follow-up outcomes as means and standard deviations. We used analysis of covariance to compare outcomes between groups at 17 weeks adjusting for baseline scores. Results are reported as both a mean between-group difference and Cohen's *d* effect size each with 95% CIs. We examined the effect of missing data by imputing missing follow-up data using chained equations (White *et al.* 2011) incorporating data for all relevant variables. We then used the imputed dataset (using 30 imputations) to compare groups taking account of the multiply imputed data (White *et al.* 2011). We used procedures of Jacobson & Truax (1991) for calculating reliable and clinically significant change to quantify clinical improvement in depressive symptoms on the EPDS (Matthey, 2004). Statistical analyses were performed using SPSS v. 18 (IBM, USA) and Stata v. 12 (StataCorp LP, USA).

The study was reviewed and received ethical approval from the University of Exeter ethics review committee.

## Results

### Recruitment and attrition

**Fig. 1.**
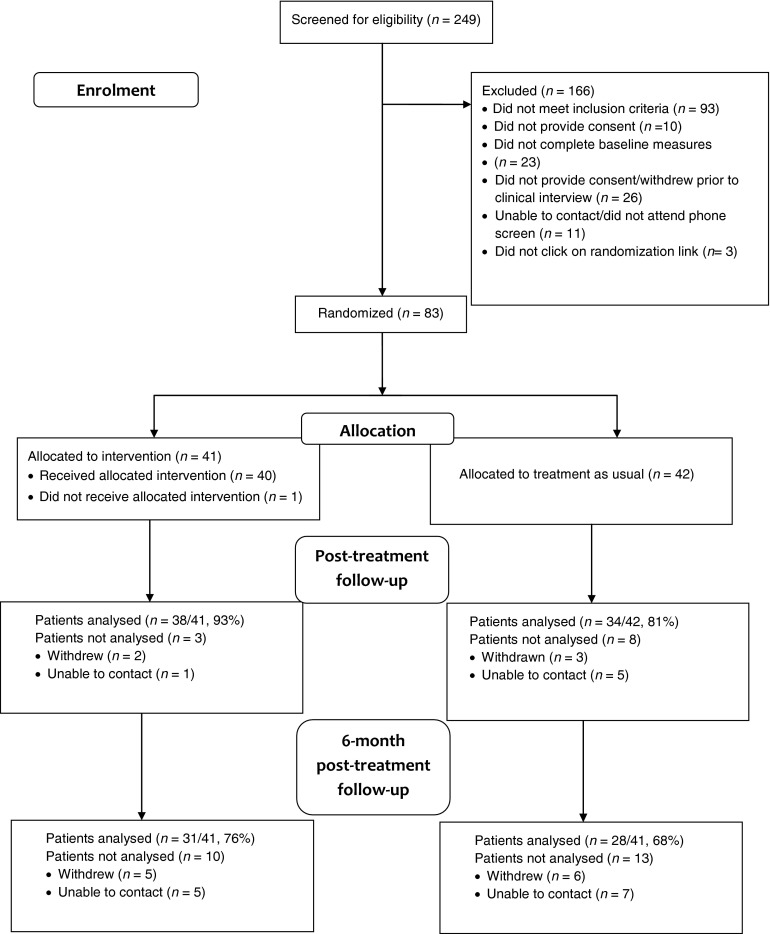
CONSORT 2010 flow diagram.

Fig. 1 presents the CONSORT (CONsolidated Standards of Reporting Trials) diagram showing the flow of participants through the trial. Demographic characteristics are given in Table 2. Of the 249 women who expressed initial interest in the trial, we randomized 83 women (33%): 41 to NetmumsHWD and 42 to TAU. Post-treatment EPDS was completed by 37/41 (90%) women in the NetmumsHWD condition and by 34/42 (81%) women in the TAU group (*χ*^2^ = 1.45, *p* = 0.23). A 6-month follow-up EPDS was completed by 31/41 (76%) women in the NetmumsHWD group and 28/41 (68%) women in the TAU group (*χ*^2^ = 0.37, *p* = 0.47). There were no differences between those who expressed an interest in the trial, were randomized, completed the post-treatment or 6-month follow-up assessment measures and those who did not on baseline measures of psychological functioning or by sociodemographic characteristics.

**Table 2.** Characteristics of participants at baseline

Baseline characteristic	NetmumsHWD	TAU
Income, *n* (%)		
<£10000	4 (9.2)	2 (4.8)
£10000–£19000	6 (14.6)	10 (23.8)
£20000–£29999	6 (14.6)	4 (9.5)
£30000–£39999	5 (12.2)	10 (23.8)
£40000–£49999	4 (9.8)	7 (16.7)
£50000–£59999	6 (14.6)	2 (4.8)
£60000–£69999	7 (17.1)	3 (7.1)
£70000–£79999	2 (4.9)	3 (7.1)
⩾£80000	–	1 (2.4)
Work status, *n* (%)		
Homemaker/maternity leave/disability	32 (80.5)	32 (80.5)
Full- or part-time employment	3 (7.3)	6 (14.3)
Student or volunteer	3 (7.3)	4 (9.6)
Relationship status, *n* (%)		
Married/cohabiting	38 (92.6)	38 (90.5)
Not in a relationship now	3 (7.3)	4 (9.5)
Academic qualifications, *n* (%)		
None	1 (2.4)	n.a.
Secondary	4 (9.7)	11 (26.2)
Post-16 years	13 (31.7)	10 (23.8)
Undergraduate degree	13 (31.7)	12 (28.6)
Graduate degree	10 (24.4)	9 (21.4)
Number of children, *n* (%)		
1	19 (46.3)	16 (38.1)
2	18 (43.9)	16 (38.1)
3	1 (2.4)	9 (21.4)
4 or more	3 (7.6)	1 (2.4)
Ethnic group, *n* (%)		
White/British	38 (92.6)	39 (92.9)
Asian	1 (2.4)	n.a.
Mixed white/African/Caribbean	n.a.	2 (4.8)
African	n.a.	1 (2.4)
Other	2 (4.8)	n.a.

NetmumsHWD, Netmums Helping With Depression; TAU, treatment as usual; n.a., not applicable.

### Treatment adherence

**Fig. 2.**
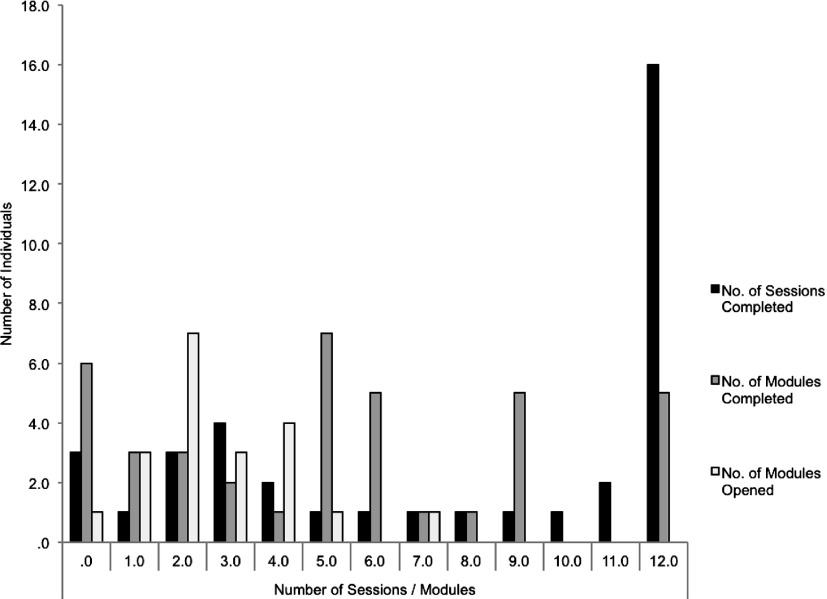
Frequencies of the number of sessions/modules completed or opened.

Choice of optional modules was relatively evenly distributed across the different modules, although the most frequently chosen module was ‘Being a good enough mother’ (22%, *n* = 18/82; denominator 82 = possible module choices = 41 women in treatment × two module choices). Women viewed a mean of 6.74 (s.d. = 4.53) computer sessions and completed 5.36 (s.d. = 4.62). A total of 11 women (5%) completed eight or more computer sessions. Of these, five (1.9%) women completed 12 computer sessions. The mean number of completed telephone support sessions was eight (s.d. = 4.5, mode = 12). Telephone session 1 had a mean duration of 50 min (s.d. = 4.31), and sessions 2–12 had a mean duration of 29 min (s.d. = 4.76). The average total time of sessions per participant was 253 min. See Fig. 2 for detailed frequencies of number of telephone support and Internet modules completed. Although unexpected missed appointments were infrequent (mean = 0.35, s.d. = 0.63), patient appointment cancellations and rescheduling were more common, and were typically communicated via text or email (mean *n* = 1.65, s.d. = 0.78). Of four women who discontinued treatment and provided reasons for doing so, two reported they did not have enough time, one stated she was overwhelmed, and one reported feeling better.

### Predictors of treatment adherence

Participants were from a wide socio-economic and demographic range (Table 3), with high levels of depressive symptoms [EPDS mean for intervention 20.24 (s.d. = 3.28); TAU 21.07 (s.d. = 4.0)] and functional impairment [mean WASAS for intervention 29.8 (s.d. = 7.8); TAU 28.8 (s.d. = 9.1)]. Table 3 shows analysis of predictors of treatment adherence. There were no predictors of number of telephone sessions completed. Women with lower perceived support and who were working or studying for a degree completed fewer modules. Lastly, women with poorer WASAS baseline functioning and who were of a lower socio-economic status (SES) opened fewer sessions than women with higher functioning or SES level.

**Table 3.** Predictors of treatment adherence

Factor	Trial adherence		Telephone sessions		Modules completed		Modules opened	
	Test statistic: *t*	*p*	Test statistic: *r* correlation	*p*	Test statistic: *r* correlation	*p*	Test statistic: *r* correlation	*p*
EPDS	−0.59	0.56	−0.01	0.94	−0.26	0.10	0.20	0.41
GAD-7	−0.53	0.60	−0.16	0.35	0.00	0.98	0.20	0.20
WASAS	−0.44	0.67	−0.28	0.09	−0.15	0.35	0.66	0.01
SPS	−10.32	0.34	−0.01	0.96	0.33	0.04	0.16	0.49
PBQ	−10.64	0.52	0.11	0.51	0.07	0.67	0.38	0.10
Income level	Eta	0.12	−0.01	0.97	−0.52	0.02	−0.52	0.02
			Goodman & Kruskal lambda		Goodman & Kruskal lambda		Goodman & Kruskal lambda	
Academic qualification	−0.65	0.52	0.05	0.86	0.22	0.16	0.08	0.70
Relationship status	0.20	0.35	0.04	0.31	0.09	0.26	0.08	0.65
Number of children	−0.58	0.11	0.26	0.12	0.27	0.45	0.09	0.31
Work status	0.07	0.95	0.17	0.17	0.22	0.04	0.23	0.24

EPDS, Edinburgh Postnatal Depression Scale; GAD-7, Generalized Anxiety Disorder Screener; WASAS, Work and Social Adjustment Scale; SPS, Social Provision Scale; PBQ, Postnatal Bonding Questionnaire.

### Symptoms of depression, anxiety and social adjustment

Mean differences and between-subject effect sizes for the symptom measures are shown in Table 4. There was a between-group difference in EPDS and GAD-7 scores for the observed data analysis at post-treatment favouring the NetmumsHWD group. These differences in the observed results correspond to large Cohen's *d* effect sizes of EPDS (−0.87, 95% CI −0.42 to −1.32) and GAD-7 (−0.59, 95% CI −1.11 to −0.07). There was also a between-group difference in the observed WASAS scores favouring the NetmumsHWD group corresponding to a moderate effect size (−0.57, 95% CI −0.07 to 1.11). There were no between-group differences in postnatal self-reported bonding or perceived support scores between women in the NetmumsHWD group and those in the TAU group, reflecting a small (0.29, 95% CI −0.80 to −0.22) and medium (0.50, 95% CI 1.02 to −0.02) effect size, respectively. In the multiple imputation sensitivity analyses, EPDS, GAD-7 and WASAS scores at 17 weeks post-randomization remained in favour of NetmumsHWD. At 6 months post-treatment follow-up, there was a trend favouring NetmumsHWD (mean = 8.26, s.d. = 5.50) over TAU (mean = 11.14, s.d. = 6.35; mean difference −2.69, 95% CI −5.80 to −0.42), corresponding to a large effect size (−0.78, 95% CI −1.82 to 0.10). This was replicated in the multiple imputation sensitivity analysis (mean difference −2.28, 95% CI −5.41 to 0.84), corresponding to a large effect size (−0.678, 95% CI −1.121 to −0.236).

**Table 4.** Means, s.d. and effect sizes (Cohen's d) including 95% CIs for the EPDS for observed and multiple imputation analyses; WASAS and the GAD-7 for observed and imputed analyses

Condition	Measurement	Pre-treatment		17-week post-randomization		Between-group difference*	*p*	Effect size
		Mean (s.d.)	*n*	Mean (s.d.)	*n*	Mean (95% CI)		Cohen's *d* (95% CI)
Intervention	EPDS	20.24 (3.28)	41	11.05 (4.71)	37	−3.02 (−5.34 to −0.70)	0.01	−0.87 (−1.32 to −0.42)
Control		21.07 (4.0)	42	14.26 (5.11)	34	Imputed −3.26 (−5.57 to –0.95)	Imputed <0.01	Imputed −0.960 (−1.415 to −0.505)
Intervention	GAD-7	13.90 (3.82)	41	8.71 (4.61)	31	−2.74 (−5.19 to −0.30)	0.03	−0.59 (−1.11 to 0.07)
Control		14.12 (4.78)	42	11.29 (5.49)	28	Imputed −2.73 (−4.98 to −0.49)	Imputed 0.02	Imputed −0.655 (−1.097 to −0.213)
Intervention	WASAS	19.24 (4.90)	41	13.13 (6.70)	31	−3.67 (−7.61 to −0.17)	0.04	−0.57 (1.10 to −0.06)
Control		19.64 (6.10)	42	17.18 (7.25)	28	Imputed −4.01 (−7.24 to −0.78)	Imputed 0.02	Imputed −0.756 (1.202 to −0.310)
Intervention	PBQ	31.66 (15.74)	41	22.57 (12.99)	31	1.42 (−4.32 to 7.16)	0.62	0.29 (0.22 to 0.80)
Control		24.93 (18.72)	42	17.57 (11.17)	28	Imputed 1.18 (−4.77 to 7.13)	Imputed 0.69	Imputed 0.247 (−0.185 to 0.679)
Intervention	SPS	67.61 (9.71)	41	73.63 (9.83)	31	2.28 (−1.79 to 2.03)	0.27	0.50 (−0.02 to 1.02)
Control		67.36 (10.89)	42	68.39 (10.49)	28	Imputed 2.44 (−1.62 to 6.51)	Imputed 0.23	Imputed 0.25 (−0.179 to 0.686)

s.d., Standard deviation; CI, confidence interval; EPDS, Edinburgh Postnatal Depression Scale; GAD-7, Generalized Anxiety Disorder Screener; WASAS, Work and Social Adjustment Scale; SPS, Social Provision Scale; PBQ, Postnatal Bonding Questionnaire.

* Difference in post-treatment (completer) score between groups adjusting for pre-treatment score.

### Reliable and clinically significant improvement

A reliable and clinically significant improvement in depression scores was seen in 62.2% (*n* = 23/37) of those in the NetmumsHWD group at post-treatment compared with 29.4% (*n* = 10/34) of those in the TAU group. After adjustment for baseline EPDS the odds ratio for a reliable and clinically significant improvement in the treatment group compared with control was 0.26 (95% CI 0.10–0.71).

### Health service utilization

Health service utilization is presented in Table 5. There were no differences in utilization between the NetmumsHWD and TAU groups at baseline or at follow up. At baseline, 63% (52/83) of the total randomized sample was taking an antidepressant medication, and although none of the women randomized were currently in psychological treatment for depression, 37% (25/83) had received an average of 1.34 (s.d. = 3.85) sessions of out-patient treatment in the previous 3 months.

**Table 5.** Health service utilization at baseline^a^ and 17-week follow-up by treatment condition

	NetmumsHWD		TAU		*p*
	Mean (s.d.)	*n* (%)	Mean (s.d.)	*n* (%)	
Childbirth hospital					
Baseline	1.81 (3.40)	16/41 (39)	1.31 (4.46)	8/42 (19)	0.57
17-week follow-up	0.10 (0.62)	1/30 (2.4)	0.64 (3.11)	2/27 (7.4)	0.28
Maternal general health hospital					
Baseline	0.05 (0.22)	2/41 (4.9)	0.17 (0.66)	4/42 (9.5)	0.28
17-week follow-up	0	0/30	0	0/27	n.a.
Infant hospital					
Baseline	1.17 (3.26)	12/41 (29.3)	0.88 (2.01)	11/42 (26.2)	0.63
17-week follow-up	0.24 (0.99)	4/30 (13.3)	0.38 (1.35)	6/27 (22.2)	0.60
Mental health hospital					
Baseline	0	0/41	0.05 (0.31)	1/42 (2.4)	0.11
17-week follow-up	0	0/30	0.05 (0.31)	1/27 (2.4)	0.33
Mental health out-patient^b^					
Baseline	1.68 (4.57)	15/41 (36.6)	1.02 (3.02)	10/42 (37)	0.44
17-week follow-up	5.07 (3.34)	14/30 (46.7)	10.37 (15.76)	11/27 (40.7)	0.23
Health community service					
Baseline	7.51 (7.89)	39/41 (95.1)	6.95 (6.25)	40/42 (95.2)	0.72
17-week follow-up	3.32 (3.94)	28/30 (93.3)	2.95 (3.31)	25/27 (92.6)	0.65
Antidepressant medication					
Baseline	2.95 (0.77)	27/41 (65.9)	3.07 (0.75)	23/42 (59.5)	0.47
17-week follow-up	2.96 (0.76)	19/27 (70.4)	3.07 (0.75)	19/30 (63.3)	0.38

NetmumsHWD, Netmums Helping With Depression; TAU, treatment as usual; s.d., standard deviation; n.a., not applicable.

a Health care utilization at baseline assessed use in the previous 6 months.

b Mental health utilization was in the 6 months previously. Women were excluded from the trial if they were currently receiving mental health therapy.

## Discussion

We found that we were able to recruit and retain sufficient participants to the study and that the supported NetmumsHWD programme had high levels of trial and clinical adherence, demonstrating promise as a feasible treatment for PND. The between-group effect size of the NetmumsHWD programme was large (−0.87, 95% CI 0.42–1.32), and the effects were reliably and clinically significant for over 60% of users. Although the 95% CI was wide, spanning a medium to very large effect size, these results compare favourably with effect sizes from meta-analyses of face-to-face treatments for PND (−0.65, 95% CI −0.45 to −0.86; Sockol *et al.* 2011) and with online CBT treatments for MDD that provide an element of guided support (0.61, 95% CI 0.45–0.77; Andersson & Cuijpers, 2009), including a recent trial of guided online CBT for general maternal depression (Sheeber *et al*. 2012). These results suggest that the NetmumsHWD programme is feasible, and may offer promise as an effective treatment for PND. As the NetmumsHWD programme requires minimal therapist time (average session time was 29 min; average total time of sessions per participant was 253 min), it may offer a cost-effective and accessible treatment option for PND.

We recruited and randomized 33% of women who expressed interest in the study. We would therefore expect similar rates of recruitment in a future trial. We retained 83% of women in the trial at post-treatment follow-up and 71% at 6-month follow-up, although it was more difficult to retain women in the TAU condition. These short-term attrition rates compare favourably with other face-to-face and online treatment trials (Kaltenthaler *et al.* 2008; Christensen *et al.* 2009; Andrews *et al.* 2010). Further qualitative work with women in this study may highlight modifiable factors contributing to attrition. However, we note that these trial attrition rates compare very favourably with another recent online trial of BA for PND that did not include telephone guidance (O'Mahen *et al.* 2013*b*). In that trial, we retained 38% (*n* = 343/910) of women, a rate consistent with other unsupported, online treatment trials (Etter, 2005; Farvolden *et al.* 2005; Christensen *et al.* 2009). Women completed an average of five Internet sessions (s.d. = 4.76) and a mode of 12 telephone sessions. Many women began to incorporate content from the optional modules when they worked on their first functional analysis and alternate plan in the initial core sessions. Although the Internet sessions were designed to be completed in 1 week, there was considerable variability amongst women in the number of weeks it took them to complete each Internet session. Having a higher income, being on maternity leave or not working, having higher perceived social support, and better social and occupational functioning at baseline were related with opening and completing more modules. Women with complex life circumstances required more time to work through materials, and supporters helped them to appropriately pace and utilize the treatment content. Overall, the higher rates of treatment and trial adherence retention we achieved with guided support in this trial are in line with a growing body of research demonstrating that the provision of guidance in online treatments enhances treatment uptake and retention (Cuijpers *et al.* 2010).

The results suggest that NetmumsHWD offers promise in improving functioning and reducing anxiety relative to women on TAU. The effect sizes were moderate in size. Although there was a trend in the NetmumsHWD programme towards improved perceived support and maternal self-reported bonding with infant, the effect sizes were small. In all analyses, the CIs were wide, which we would expect in a sample of this size. The broader literature in this area suggests that women with support and/or bonding problems may require specific interventions directed at remediating these difficulties (Poobalan *et al.* 2007). Furthermore, because previous research has demonstrated that not all mothers with PND experience bonding difficulties with their infants (Tharner *et al.* 2012), it may be important to direct specific interventions only to those mothers who are experiencing difficulties. A larger, definitive trial would permit examinations of whether the modules on communication and parenting are helpful for women experiencing difficulties in these specific domains.

### Implications

The results from this trial suggest that the provision of guidance in an Internet-based treatment for PND is associated with less trial and treatment attrition. The intention-to-treat imputed effect sizes of the NetmumsHWD intervention contrasted with TAU are comparable with the intention-to-treat effects for completers only in a previous unsupported trial of this intervention (O'Mahen *et al.* 2013*b*), but the key difference in this trial is that we had considerably less attrition. Importantly, results from the previous trial of PND and other studies of unsupported online treatments (Christensen *et al.* 2004, 2009; O'Mahen *et al.* 2013*b*) suggest that some individuals do not require guidance to persist with treatment. Our preliminary findings from this study also point to subgroups of individuals who may require more support. Research investigating psychological and sociodemographic factors associated with treatment adherence may support stratified, nuanced treatments that personalize levels of support.

Further, it is unclear from the results in the current trial whether the effect of the intervention was due to the computer content itself, the telephone-based support, or an interaction of the two. Multifactorial designs comparing unsupported and supported versions of the treatment may help to explicate the active components of the interventions.

### Limitations

A large-scale examination of a guidance-based version of the NetmumsHWD treatment is needed to definitively assess its efficacy and cost-effectiveness. Future research would benefit from longer-term follow-up, clinical interview assessment of mood at post-treatment, and observational assessments of mother–child interaction. Internet treatments may serve as a treatment endpoint, or may be a starting point leading to further treatment. For example, we noted that some women in treatment also struggled with post-traumatic stress disorder and grief, in addition to MDD, and some initiated new treatments focused on their co-morbidities alongside NetmumsHWD. It is important to demonstrate how the cost of Internet treatment fares relative to other treatment provision. A large-scale trial would also allow for the examination of definitive predictors of treatment adherence. Based on the baseline and post-17-week correlations and minimum clinically important difference data from the larger literature, the sample size calculations for a larger trial would range from 69 per group, when using a power of 80% and 20% attrition, to 132 per group when using 90% power and 30% attrition. Lastly, it is important to note that Internet interventions may not be appropriate for all individuals. For example, users of the current trial were all Netmums users who may have found Internet approaches more acceptable.

## Conclusion

Our study adds to a growing body of literature that has demonstrated the feasibility and clinical utility of Internet interventions for depression. To our knowledge, this is the first RCT demonstrating the clinical potential of a supported online intervention specifically developed for PND. Such an intervention may hold promise for reaching groups of women who do not have local access to empirically supported treatments, struggle with issues of stigma, or have a particular need for flexibility and in-home treatment delivery (Kaltenthaler *et al.* 2006).

## Footnotes

† The notes appear after the main text.

## Notes

1 All effect sizes reported are either Cohen's *d* or Hedges' *g*. Hedges' *g* is comparably similar to Cohen's *d* but includes statistical adjustments for small sample size (Rosenthal & Rosnow, 2008).
